# Self-Efficacy and Emotional Intelligence as Predictors of Perceived Stress in Nursing Professionals

**DOI:** 10.3390/medicina55060237

**Published:** 2019-06-01

**Authors:** María del Mar Molero Jurado, María del Carmen Pérez-Fuentes, Nieves Fátima Oropesa Ruiz, María del Mar Simón Márquez, José Jesús Gázquez Linares

**Affiliations:** 1 Department of Psychology, Faculty of Psychology, University of Almería, 04120 Almería, Spain; mmj130@ual.es (M.d.M.M.J.); foropesa@ual.es (N.F.O.R.); msm112@ual.es (M.d.M.S.M.); 2 Department of Psychology, Universidad Autónoma de Chile, Región Metropolitana, Providencia 7500000, Chile; jlinares@ual.es

**Keywords:** health behavior, worksite health promotion, quantitative methods, training health professionals, worksite health

## Abstract

*Background*: Nursing professionals face a variety of stressful situations daily, where the patients’ own stresses and the demands of their family members are the most important sources of such stress. *Methods*: The main objectives pursued were to describe the relationships of self-efficacy and emotional intelligence with perceived stress in a sample of nursing professionals. We also developed predictive models for each of the components of perceived stress based on the dimensions of emotional intelligence and self-efficacy, for the total sample, as well as samples differentiated by sex. This study sample consisted of 1777 nurses and was conducted using multiple scales: the perceived stress questionnaire, general self-efficacy scale, and the brief emotional intelligence survey for senior citizens. *Results*: The variables stress management, mood, adaptability, intrapersonal skills, and self-efficacy explained 22.7% of the variance in the harassment–social component, while these same variables explained 28.9% of the variance in the irritability–tension–fatigue dimension. The variables mood, stress management, self-efficacy, intrapersonal, and interpersonal explained 38.6% of the variance in the energy–joy component, of which the last variable offers the most explanatory capacity. Finally, the variables stress management, mood, interpersonal, self-efficacy and intrapersonal skills explained 27.2% of the variance in the fear–anxiety dimension. *Conclusion*: The results of this study suggest that one way to reduce stress in professionals would be to help them improve their emotional intelligence in programs (tailored to consider particularities of either sex) within the framework of nursing, enabling them to develop and acquire more effective stress coping strategies, which would alleviate distress and increase the wellbeing of health professionals.

## 1. Introduction

Stress is defined as a complex psychobiological process which is experienced when the individual perceives a threat or danger in the environment [1]. When we think of stress, we immediately associate it with lack of availability of personal and social resources for coping with challenges [2]. Health professionals have shown higher levels of psychosocial stress than other population samples [3]. Nursing professionals face a variety of stressful situations daily, where the patients’ own stresses and the demands of their family members are the most important sources of such stress [4]. These circumstances could well affect their professional competence, considering that nursing professionals have been described as a group especially susceptible to burnout [5].

The theoretical focus on stress proposed by Levenstein et al. [6] has its roots in psychosomatic research and places emphasis on cognitive perceptions associated with stress, instead of the emotional states or the effects of life events. A lower level of perceived stress in the context of health has been associated with higher scores on emotional intelligence [7,8,9,10]. Emotional intelligence is a psychological construct introduced by Salovey and Mayer [11] in the field of education and developed by Goleman [12] and Bar-On [13] with important repercussions, both theoretical and practical, in different areas of psychology (organizational, educational, health). For example, Bar-On referred to emotional intelligence as a variety of noncognitive skills, competencies, and abilities that influence a person’s capacity to succeed in the face of daily demands and pressures. Being emotionally intelligent implies the ability to address, understand, and feel one’s own emotions and those of others, and being able to respond and act accordingly (intrapersonal, interpersonal, stress management, adaptability, and general mood).

Recent research has revealed that, specifically in nursing professionals, emotional intelligence protects from burnout [14], predicts commitment to the job [15], and is related to job satisfaction [16] and wellbeing [17,18,19], as well as with problem-solving and perceived competence [18,19]. Emotional intelligence also positively influences attention focused on the patient [20,21]. Therefore, an emotionally intelligent person could cope better with work on the job and also be more attentive, often in clinical decision-making [22]. 

In the scope of healthcare, research reveals that female nurses show higher levels of emotional intelligence in the “interpersonal” dimension [15,23], while other studies have found that male nurses score higher in the “stress management” dimension [23,24]. Nevertheless, results have also been found in both directions in the “intrapersonal” dimension of emotional intelligence, that is, with higher scoring female nurses [23,24], and similarly, with the “mood” dimension, which has sometimes had higher scoring male nurses [24], while others showed higher scoring female nurses [15]. In addition to analysis of emotional intelligence components based on meta-analyses [25], it is suggested that a high level of the construct is associated with the use of more efficient emotional regulation strategies. Therefore, higher scores in emotional intelligence indicate that those nursing professionals are able to minimize the intensity and presence of negative emotions, which consequently, have a positive repercussion on management of stressful situations and protect against burnout. Along this line, the role of emotional intelligence as a moderator in the relationship between negative emotions and burnout is proposed [26]. Meta-analyses have also previously corroborated the relationships between emotional intelligence based on capacity and job performance [27]. One of the main findings in this respect refers to inconsistencies based on the predictive value of emotional intelligence depending on the characteristics of the position, showing positive value in jobs with high emotional load and negative value in those with a low emotional load.

Furthermore, general self-efficacy, understood as the perception of competence in resolving stressful situations, in view of the results of meta-analyses, has also been considered a moderating variable in stress and protection from burnout at work [28]. In other empirical studies, where risk and protection of burnout in healthcare personnel have been analyzed, data have suggested a significant negative relationship with all the components of emotional intelligence, self-efficacy and perceived social support [14]. Therefore, it may be said that the risk of burnout is greater in persons with low scores in general self-efficacy and emotional intelligence, emphasizing the value of stress management as the most notable protective factor against the tendency to suffer from burnout. Along this line, in the study by Bodys-Cupak et al. [29], the feeling of self-efficacy had a significant impact on the level of stress of Polish nursing students, as well as the way they coped with more difficult situations. In a longitudinal study conducted with an adult population in China, general self-efficacy was also negatively correlated with stress [30]. In some studies, the pattern of type A behavior has been associated with a high level of stress [31], and type A personality and self-efficacy with burnout [32,33]. In addition, the relationships between self-efficacy and personality have remained stable over different samples and cultures [34]. Therefore, general self-efficacy is also an important variable to be considered in the relationship between personality and perceived stress [35,36]. Specifically, negative associations have been found with perceived stress and extraversion, conscientiousness, friendliness and openness, while neuroticism had a positive relationship [35]. More recently, results have been found supporting a significant association between neuroticism and extraversion with perceived stress and general self-efficacy [36]. In both cases, the mediating role of general self-efficacy in the relationship between personality factors and perceived stress is emphasized. 

Based on these empirical findings and being convinced that emotional intelligence and self-efficacy can reduce the negative consequences of stress, the following study on perceived stress and its relationship with self-efficacy and emotional intelligence was designed. The main objectives pursued were (1) describing the relationships of self-efficacy and emotional intelligence with perceived stress in a sample of nursing professionals; while (2) developing predictive models for each of the components of perceived stress based on the dimensions of emotional intelligence and self-efficacy, for the total sample and differentiated by sex. 

Keeping in mind the results of prior research, the following general research hypotheses were posed: (1) there is a significant negative relationship between emotional intelligence and perceived stress, as well as between self-efficacy and stress in nursing professionals; and (2) emotional intelligence and self-efficacy are important predictors of perceived stress. 

## 2. Materials and Methods

### 2.1. Participants

The initial sample was made up of 1883 nurses from Andalucía (Spain) who were randomly selected from various centers. We identified 106 cases that were removed from the sample for not completing the entire questionnaire (19 subjects) or because we found that they had completed it randomly (87 subjects). The final sample consisted of 1777 nurses in Andalucía (Spain), all of them in active employment at the time of data collection (71.6% with temporary contracts *n* = 1273, and 28.4% with permanent contracts *n* = 504).

The mean age of the participants was 32.02 (SD = 6.69) years, in a range of 22 to 60 years. Distribution of the sample by sex was 85.4% (*n* = 1517) women and 14.6% (*n* = 260) men, with a mean age of 32.01 (SD = 6.63) and 32.10 (SD = 7.01) years, respectively. Their marital status was 51.5% (*n* = 916) single, 46.1% (*n* = 819) married, 2.3% (*n* = 40) divorced or separated, and 0.1% (*n* = 2) widowed participants.

### 2.2. Instruments 

The Perceived Stress Questionnaire by Levenstein et al. [6] was specifically designed to measure stress in psychosomatic clinical research. The original version had 30 items distributed in six scales: harassment–social acceptance (“You feel lonely or isolated”), overload (“You have too many things to do”), irritability–tension–fatigue (“You are irritable or grouchy”), energy–joy (“You are full of energy”), fear–anxiety (“You feel loaded down with responsibility”), and self-realization–satisfaction (“You feel you’re doing things you really like”). Authors such as Sanz-Carrillo, García-Campayo, Rubio, Santed, and Montoro [37], found general reliability of 0.80 in a study sample of healthcare workers and students. In this case, the alpha coefficient was 0.85 (harassment–social acceptance (0.83), overload (0.69), irritability–tension–fatigue (0.88), energy–joy (0.80), fear–anxiety (0.62), and self-realization–satisfaction (0.60)). 

The General Self-Efficacy Scale [38] measure, which consists of ten items with a four-point Likert-type response scale, evaluates a person’s own perception of his/her competence in handling stressful situations effectively. Authors such as Sanjuán, et al. [39] analyzed the scale’s reliability, finding a Cronbach’s alpha of 0.87. In this study, based on the calculation of the scale’s internal consistency, the alpha was 0.91.

The Brief Emotional Intelligence survey for senior citizens (EQ-i-20M) [40], validated and scaled by the authors for an adult Spanish population, was adapted for adults from the Emotional Intelligence Inventory: Young Version (EQ-i-YV) by Bar-On and Parker [41]. It consists of 20 items with four answer choices arranged on a Likert-type scale. It is structured in five variables: intrapersonal, interpersonal, stress management, adaptability, and mood. The Cronbach’s alpha found for each of the scales is 0.90 for intrapersonal, 0.75 for interpersonal, 0.83 for stress management, 0.82 for adaptability, and 0.88 for mood.

### 2.3. Procedure

This study was approved by the Bioethics Committee of the University of Almeria (Ref: UALBIO2017/011). Prior to collecting data, compliance with the standards of participant information, confidentiality and ethical data processing were ensured. The questionnaires were implemented on a web platform which enabled participants to fill them out online. A series of control questions were included to detect random or incongruent answers and discard them from the study sample.

### 2.4. Data Analysis

First, the relationships between variables were checked by bivariate correlation analysis. To find out how the predictor variables (self-efficacy (SELF), and emotional intelligence dimensions: intrapersonal (INTRA), interpersonal (INTER), stress management (STRESS_M), adaptability (ADAPT), and mood (MOOD)) were related to the criterion variables (perceived stress: harassment–social acceptance (H–SA), overload (OVER), irritability–tension–fatigue (I–T–F), energy–joy (E–J), fear–anxiety (F–A), self-realization–satisfaction (SR–R)), multiple linear regression (stepwise) analyses were performed for both the total sample and for each of the groups by sex.

Then a simple mediation analysis was performed for each component of perceived stress, taking self-efficacy as the predictor variable and stress management as the mediator. The SPSS macro for mediation models was used for this [42], applying bootstrapping with coefficients estimated from 5000 bootstraps.

## 3. Results

### 3.1. Emotional Intelligence, Self-Efficacy and Perceived Stress

As observed in Table 1, self-efficacy correlated negatively with most of the perceived stress components (H–SA; I–T–F; F–A; SR–S) and had a positive correlation with energy–joy (E–J).

The relationships between the components of perceived stress and the dimensions of emotional intelligence observed were as follows: H–SA, was negatively correlated with intrapersonal, interpersonal, stress management, adaptability, and mood. Overload was negatively correlated with intrapersonal, stress management, and mood. The I–T–F component was negatively correlated with all the dimensions of emotional intelligence. On the contrary, E–J had positive correlations in all cases. Furthermore, the F–A component was negatively correlated with all the dimensions of emotional intelligence. Finally, SR–S also correlated negatively in all cases.

### 3.2. Predictors of the Harassment–Social Acceptance Dimension of Perceived Stress

Based on the data from the correlation analyses, multiple linear regression analyses were performed to identify the predictor variables in each case. 

According to the data for the H–SA component shown in Table 2, the regression analysis produced five variables, of which the last offers the most explanatory capacity, with 37% of the variance explained by the variables included in the model. To confirm the validity of the model, residual independence was analyzed. The Durbin–Watson value was *D* = 1.93, confirming the absence of positive or negative self-correlation. Furthermore, *t* was associated with the probability of an error below 0.05 in all the variables included in the model, while the standardized coefficients revealed that the variable which had the highest explanatory value was mood. Finally, according to the tolerance and VIF, absence of collinearity of variables in the model may be assumed.

**Figure 1:**
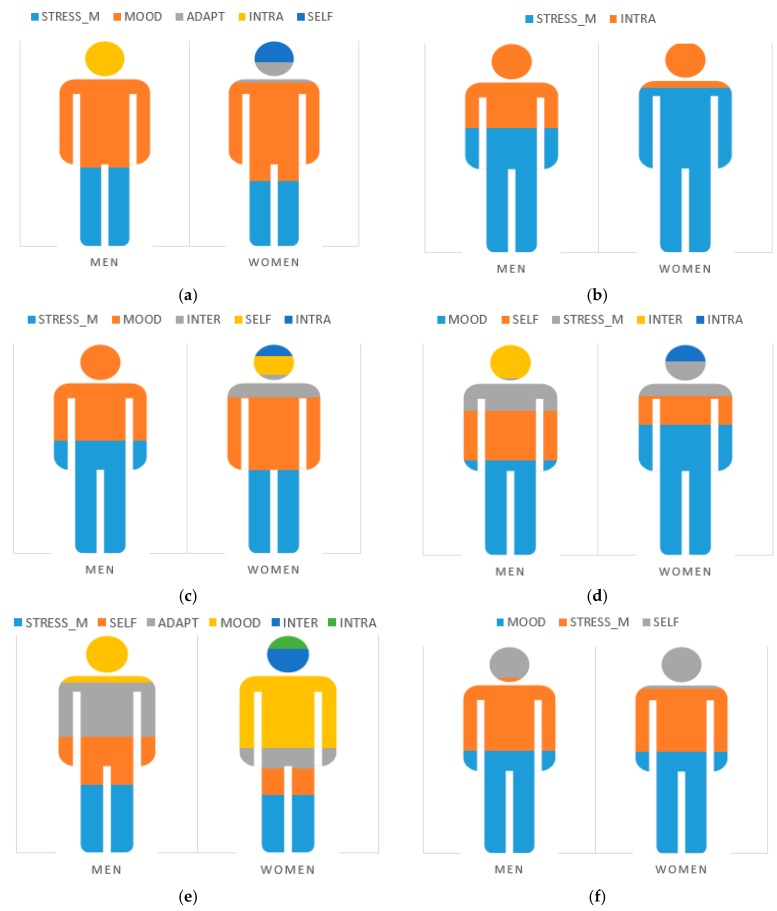
Stepwise multiple linear regression model by sex. (**a**) Harassment–social acceptance; (**b**) Overload; (**c**) Irritability–tension–fatigue; (**d**) Energy–joy; (**e**) Fear–anxiety; (**f**) Self-realization–satisfaction.

Figure 1 shows the regression analysis models for the H–SA dimension, taking sex as the selection variable. For the male group, the regression model explained 32.4% of the variance (*R*^2^ = 0.32), while for the female group, the model explained 38.2% (*R*^2^ = 0.38) of the variance. In both cases, mood was the strongest predictor. 

### 3.3. Predictors of the Overload Dimension of Perceived Stress

Two variables were found for the overload component of perceived stress, the second of which explained 6.5% of the variance. In this case, the Durbin–Watson *D* value confirmed model validity (*D* = 1.98). The *t* statistic detected the probability of association with an error below 0.05, for all the variables included in the model. According to the standardized coefficients found, stress management was the strongest predictor of overload. Based on tolerance and the VIF values found, absence of collinearity of variables may be assumed. 

Figure 1 shows the regression analysis results for the overload dimension taking sex as the selection variable. For men, the regression model explained 6.8% of the variance (*R*^2^ = 0.06), while for women, the model explained 6.6% of the variance (*R*^2^ = 0.06). In both cases, stress management was the strongest predictor. 

### 3.4. Predictors of the Irritability–Tension–Fatigue Dimension of Perceived Stress

For the I–T–F dimension, the regression analysis produced five variables (Table 2), of which the last one explained 37.6% of the variance, with a *D* = 1.99, confirming model validity. The *t* value detected the probability of association of variables below 0.05, for all the variables included. In this case, stress management was also the strongest predictor. According to the tolerance and VIF, absence of collinearity of the variables in the model may be assumed. 

Figure 1 shows the result of the regression analysis for the I–T–F dimension taking sex as the selection variable. The variance explained was 31.8% for men (*R*^2^ = 0.31) and 38.5% for women (*R*^2^ = 0.38). In both cases, stress management was the strongest predictor.

### 3.5. Predictors of the Energy–Joy Dimension of Perceived Stress

As observed in Table 3, the regression analysis produced five variables for the E–J component, the last of which had the most explanatory capacity with 38.6% of explained variance. The Durbin–Watson statistic was *D* = 1.94, which confirms the absence of positive or negative self-correlation. It is also observed that the *t* was associated with a probability of error below 0.05 for all the variables included in the model, while the standardized coefficients reveal that the variable with the highest explanatory value in this case was mood. Finally, absence of collinearity of the variables in the model may be assumed from the tolerance and VIF. 

Figure 1 shows the result of the regression analysis for the E–J dimension taking sex as the selection variable, which explained 43.4% of the variance for men (*R*^2^ = 0.43) and 37.9% of the variance for women (*R*^2^ = 0.37). In both cases, mood was the strongest predictor. 

### 3.6. Predictors of the Fear–Anxiety Dimension of Perceived Stress

The regression analysis found five variables for the F–A dimension (Table 3), the last of which explained 27.2% of the variance, with a *D* = 1.97, confirming model validity. The *t* showed the probability of association of variables below 0.05 in all cases. Mood was the strongest predictor in the model. Absence of collinearity among the variables may be assumed from the tolerance and VIF. 

Figure 1 shows the regression analysis models for F–A with sex as the selection variable. For men, the model explained 21.2% of the variance (*R*^2^ = 0.21), while for women it explained 28.9% (*R*^2^ = 0.28). Stress was the strongest predictor for men, while for women it was mood. 

### 3.7. Predictors of the Self-Realization–Satisfaction Dimension of Perceived Stress

Three variables were found for the SR–S component of perceived stress, the third of which had the highest percentage of explained variance with 35.5%. In this case, the Durbin–Watson *D* confirmed model validity (*D* = 1.91). The *t* detected the probability of association with an error below 0.05 for the three variables included in the model. According to the standardized coefficients, mood was the strongest predictor. Based on the tolerance and VIF, absence of collinearity of variables may be assumed. 

Finally, Figure 1 shows the results of regression analysis for SR–S, taking sex as the selection variable. For men, the regression model explained 35.9% of the variance (*R*^2^ = 0.35), while for women, the model explained 35.7% (*R*^2^ = 0.35). In both cases, mood was the strongest predictor.

Based on the results of the previous regression analyses, a simple mediation model was computed for each of the perceived stress components, where the predictor variable was self-efficacy, and in all cases, the mediator variable entered was stress management. 

**Figure 2:**
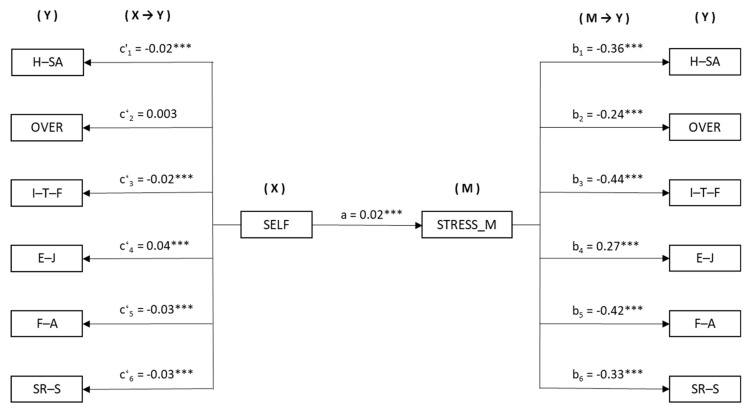
Mediation model for stress management on the relationship between self-efficacy and perceived stress components.

As shown in Figure 2, there was a significant effect of self-efficacy (X) on stress management as the mediator (*B* = 0.02, *p* < 0.001). The following regression analysis, taking each of the perceived stress components (Y) as the result variables, estimated the effect of the independent variable (in the column on the left: X→Y) and the mediator (in the column on the right: M→Y).

The results of the estimation of the direct effects, X→Y, demonstrate the significance of self-efficacy on: H–SA (*B* = −0.02 *p* < 0.001), I–T–F (*B* = −0.02 *p* < 0.001), E–J (*B* = 0.04 *p* < 0.001), F–A (*B* = −0.03 *p* < 0.001), and SR–S (*B* = −0.03 *p* < 0.001), but not on OVER, which was not significant (*B* = 0.003 *p* = 0.241). Furthermore, the M→Y effects estimated showed a significant effect of stress management (M) on all the perceived stress components: H–SA (*B* = −0.36 *p* < 0.001), OVER (*B* = −0.24 *p* < 0.001), I–T–F (*B* = −0.44 *p* < 0.001), E–J (*B* = 0.27 *p* < 0.001), F–A (*B* = −0.42 *p* < 0.001), y SR–S (*B* = −0.33 *p* < 0.001).

Finally, bootstrapping analysis of the indirect effects (X→M→Y) found significant values taking the perceived stress component as the result variable in each case: H–SA (*B* = −0.006, SE = 0.001, 95% CI (−0.011, −0.006)), OVER (*B* = −0.006, SE = 0.001, 95% CI (−0.008, −0.004)), I–T–F (*B* = −0.011, SE = 0.001, 95% CI (−0.014, −0.008)), E–J (*B* = 0.006, SE = 0.001, 95% CI (0.004, 0.009)), F–A (*B* = −0.010, SE = 0.001, 95% CI (−0.014, −0.007)), and SR–S (*B* = −0.008, SE = 0.001, 95% CI (−0.011, −0.006)).

## 4. Discussion

The main objective of this study was to explore the explanatory value of emotional intelligence and self-efficacy for perceived stress. Firstly, the literature on the relationship between these two variables (emotional intelligence and self-efficacy) and perceived stress was reviewed specifically for nursing. The most outstanding results are discussed below. 

Concerning the relationship between general self-efficacy and perception of stress, on the one hand, our results show that high scores on self-efficacy were associated with low scores in the F–A component of perceived stress, with a medium effect. That is, people who perceived themselves to be more effective experienced less stress from fear or anxiety, probably because they thought they could cope successfully with threatening situations. Our data are coherent with similar research in that perceived stress is higher the less control one has of the situation, and the lower one’s perception of self-efficacy is [14,28,29,30]. 

In the other direction, the higher the scores were in self-efficacy, the higher the scores on the E–J component of perceived stress were too, with a large effect in this case. Therefore, people who perceived themselves as more self-effective also showed higher stress related to energy–joy. These data were surprising, as we would expect to find a significant, but negative, relationship between self-efficacy and stress. It might be explained from the perspective of the mediating effect of personality variables on perceived stress. Several studies have underlined the determining role of personality in the relationship between self-efficacy and stress [31,32,33]. Thus, future research could continue to study the role of personality variables and mood on the relationship between self-efficacy and stress. 

Similarly, our results coincide with those of other authors who have found a strong negative relationship between emotional intelligence and stress [7,8,9,10]. Therefore, higher emotional intelligence would lead to a lower perception of stress by health professionals. Similarly, the stress management dimension of emotional intelligence and mood are strong predictors for both men and women. Our research revealed that the stress management dimension showed a higher explanatory value for the I–T–F, F–A components (for men), H–SA, overload, and SR–S in stress and the mood dimension showed higher explanatory value for the E–J and F–A components (for women). Other studies on the role of sex in emotional intelligence have shown that male nurses score higher in the stress management dimension of intelligence than female nurses [23,24], and data for mood can found in both directions [15,16,17,18,19,20,21,22,23,24]. These results might demonstrate that perceived stress from fear or anxiety could be associated with stress management in men and with mood in women, reflecting different ways of feeling these emotions. In any case, it is suggested that future lines of research in this area should also include contextual and gender variables. As one of the components of perceived stress, the negative relationship of overload with emotional intelligence factors should be emphasized, mainly with stress management. In this case, the regression analysis did not show self-efficacy as a significant predictive variable, even though in the literature, self-efficacy is defined as perceived competence for resolving stressful situations [14,28]. It is therefore expected that appropriate mediation models will be able to contribute new data on the relationship between self-efficacy and stress [29,30], specifically with overload perceived by the individual. Along this line, the results of the mediation analysis showed that the direct effects of self-efficacy on stress components were significant, although small magnitude. Self-efficacy did not show a significant direct effect on overload. However, by entering stress management as the mediating variable, significance had a higher effect valence for all perceived stress components, including overload. Based on such results, conclusions about the relevance of the individual’s capacity to manage stress concerning emotion management may be reached. The mediating effect shown in the relationship between self-efficacy and perceived overload on stress felt by professionals should therefore be emphasized, and thus a more detailed mediation analysis of the relationships between the variables in this study are necessary to provide data approaching the complexity of the problem of stress as perceived by healthcare professionals.

Some limitations of this study should not go without mention. In the first place, the sample was taken from nurses, so one should be prudent when generalizing them to other areas of health. In extrapolating the results, it should also be taken into consideration that the sample included more female nurses than males, a characteristic trait of the nursing profession in Spain. Another point which could be a limitation is related to the characteristics of the sample, and that is that most of the nurses in the sample had a temporary contract. These are professionals who usually change the service where they work, and in some cases with several short contracts. That is why, in this case, contextual variables (for example, type of service) were not considered stable enough, and would not reflect the usual job context of the participants, which could lead to a skewed generalization of the results in this respect. Along this line, as indicated above, future lines of research have been proposed in which personal and contextual variables are considered. In that case, special attention will be given the data collection period to avoid variability in the sample which could affect comparative analysis between different services, for example. 

Secondly, data collection was done by filling in online questionnaires, and this procedure could have been subject to social desirability by the participants in the study and have partly conditioned the results. Thirdly, another limitation of this study is its cross-sectional nature. 

At the beginning of this study, we set out a series of objectives and hypotheses which we tried to address, but as we progressed, new questions and queries arose that could lead to new lines of research in the future. On the one hand, we can continue studying the relationship between emotional intelligence and perceived stress, keeping in mind contextual and gender variables. On the other hand, we can analyze further the role of personality variables and self-esteem in the relationships established between emotional intelligence, self-efficacy and perceived stress. Finally, it should be mentioned that the results of this study suggest that one way to reduce stress in professionals would be to help them improve their emotional intelligence in programs (tailored to consider the particularities of either sex) in the framework of nursing. This would enable them to develop and acquire more effective stress-coping strategies, which would alleviate distress and increase the wellbeing of healthcare professionals. 

## 5. Conclusions

The results support our first hypothesis in that significant negative correlations are found between self-efficacy and most of the components of perceived stress, except energy–joy, where the significant correlations between these two variables were positive.

Our second hypothesis was partially validated, as the results showed that emotional intelligence explained an important percentage of the variability of perceived stress, but in no case did the beliefs about self-efficacy. Both the mood and stress management dimensions of emotional intelligence turned out to be the strongest predictors of stress. 

The mood dimension of emotional intelligence was the strongest predictor of the energy–joy stress component, explaining a higher proportion of variability for men than for women. 

Moreover, the stress management dimension of emotional intelligence, in the case of men, showed the higher explanatory value of the fear–anxiety component of stress. For women, the mood dimension of emotional intelligence better explained fear–anxiety of stress, specifically. 

In the rest of the components of perceived stress, the stress management dimension of emotional intelligence showed a higher explanatory value. Thus, the irritability–tension–fatigue component of stress explained a very similar percentage of variability in women and men. The stress management dimension of emotional intelligence showed the highest explanatory value for the harassment–social component of stress. The overload component of stress explained a similar proportion of variability in women and men. Finally, the stress management dimension of emotional intelligence was the strongest predictor of the self-realization–satisfaction component of stress. 

Based on the results, it would be feasible to establish certain risk profiles with low scores on emotional intelligence, the specific way in which each of them is more or less important depending on the perceived stress dimension referred to. From this perspective, a specific approach is possible for each element of stress, keeping in mind sex differences and the relevant role of self-efficacy. With regard to this last point, the influence of self-efficacy on the relationships between emotional and perceived stress have been examined in a mediation model. 

All of this makes the design and implantation of intervention programs directed at improving the wellbeing of healthcare professionals possible and more effective. Thus, we can work on stress prevention by taking the predictive value of each of the variables analyzed as the basis. Satisfactory results are expected from what would then be a specific integrating approach to the problem of stress, with positive personal and professional consequences for those involved. 

## Figures and Tables

**Table 1:** Self-efficacy, perceived stress and emotional intelligence. Bivariate correlations.

	1	2	3	4	5	6	7	8	9	10	11
1. SELF	-										
2. H–SA	−0.31 ^***^	-									
3. OVER	−0.02	0.49 ^***^	-								
4. I–T–F	−0.30 ^***^	0.76 ^***^	0.62 ^***^	-							
5. E–J	0.39 ^***^	−0.61 ^***^	−0.32 ^***^	−0.64 ^***^	-						
6. F–A	−0.29 ^***^	0.61 ^***^	0.47 ^***^	0.71 ^***^	−0.45 ^***^	-					
7. SR–S	−0.38 ^***^	0.69 ^***^	0.37 ^***^	0.68 ^***^	−0.58 ^***^	0.60 ^***^	-				
8. INTRA	0.30 ^***^	−0.24 ^***^	−0.10 ^***^	−0.22 ^***^	0.31 ^***^	−0.20 ^***^	−0.23 ^***^	-			
9. INTER	0.42 ^***^	−0.23 ^***^	0.03	−0.18 ^***^	0.34 ^***^	−0.14 ^***^	−0.27 ^***^	0.39 ^***^	-		
10. STRESS_M	0.20 ^***^	−0.45 ^***^	−0.24 ^***^	−0.50 ^***^	0.32 ^***^	−0.39 ^***^	−0.40 ^***^	0.10 ^***^	0.18 ^***^	-	
11. ADAPT	0.57 ^***^	−0.28 ^***^	−0.02	−0.27 ^***^	0.42 ^***^	−0.26 ^***^	−0.35 ^***^	0.45 ^***^	0.59 ^***^	0.20 ^***^	-
12. MOOD	0.49 ^***^	−0.53 ^***^	−0.14 ^***^	−0.48 ^***^	0.59 ^***^	−0.43 ^***^	−0.53 ^***^	0.41 ^***^	0.46 ^***^	0.36 ^***^	0.60 ^***^

SELF = self-efficacy; H–SA = harassment–social acceptance; OVER = overload; I–T–F= irritability–tension–fatigue; E–J = energy–joy; F–A = fear–anxiety; SR–S = self-realization–satisfaction; INTRA = intrapersonal; INTER = interpersonal; STRESS_M = stress management; ADAPT = adaptability; MOOD = mood. ^***^ *p* < 0.001.

**Table 2:** Stepwise multiple linear regression model (*n* = 1777).

**Harassment–Social Acceptance**	**Model**	***R***	***R*^2^**	**Corrected *R*^2^**		**Change statistics**							**Durbin–Watson**
						**Standard error of estimation**		**Change in *R*^2^**	**Change in *F***		**Sig. of change in *F***		
	1	0.53	0.28	0.28		0.43		0.28	699.41		0.000		1.93
	2	0.59	0.35	0.35		0.41		0.07	208.67		0.000		
	3	0.60	0.36	0.36		0.41		0.00	9.01		0.003		
	4	0.60	0.36	0.36		0.41		0.00	15.58		0.000		
	5	0.60	0.37	0.36		0.41		0.00	8.40		0.004		
	**Model 5**		**Non-standardized coefficients**				**Standardized coefficients**		***t***	**Sig.**		**Collinearity**	
			***B***		**Standard Error**		**Beta**					**Tol.**	**VIF**
	(Constant)		3.76		0.08				45.36	0.000			
	Mood		−0.35		0.02		−0.42		−16.23	0.000		0.52	1.89
	Stress management		−0.26		0.01		−0.29		−14.51	0.000		0.86	1.16
	Self-efficacy		−0.01		0.00		−0.10		−4.28	0.000		0.63	1.57
	Adaptability		0.11		0.02		0.12		4.56	0.000		0.49	2.00
	Intrapersonal		−0.04		0.01		−0.06		−2.89	0.004		0.76	1.31
**Overload**	**Model**	***R***	***R*^2^**	**Corrected *R*^2^**		**Change statistics**							**Durbin– Watson**
						**Standard error of estimation**		**Change in *R*^2^**	**Change in *F***		**Sig. of change in *F***		
	1	0.24	0.05	0.05		0.55		0.05	111.03		0.000		1.98
	2	0.25	0.06	0.06		0.55		0.00	11.83		0.001		
	**Model 2**		**Non-standardized coefficients**				**Standardized coefficients**		***t***	**Sig.**		**Collinearity**	
			***B***		**Standard Error**		**Beta**					**Tol.**	**VIF**
	(Constant)		3.37		0.08				39.22	0.000			
	Stress management		−0.23		0.02		−0.23		−10.16	0.000		0.99	1.01
	Intrapersonal		−0.06		0.01		−0.07		−3.44	0.001		0.99	1.01
**Irritability–Tension–Fatigue**	**Model**	***R***	***R*^2^**	**Corrected *R*^2^**		**Change statistics**							**Durbin–Watson**
						**Standard error of estimation**		**Change in *R*^2^**	**Change in *F***		**Sig. of change in *F***		
	1	0.50	0.25	0.25		0.47		0.25	617.98		0.000		1.99
	2	0.60	0.36	0.36		0.43		0.10	293.32		0.000		
	3	0.60	0.36	0.36		0.43		0.00	11.31		0.001		
	4	0.61	0.37	0.37		0.43		0.00	14.73		0.000		
	5	0.61	0.37	0.37		0.43		0.00	8.64		0.003		
	**Model 5**		**Non-standardized coefficients**				**Standardized coefficients**		***t***	**Sig.**		**Collinearity**	
			***B***		**Standard Error**		**Beta**					**Tol.**	**VIF**
	(Constant)		4.17		0.09				45.46	0.000			
	Stress management		−0.36		0.01		−0.38		−18.97	0.000		0.86	1.16
	Mood		−0.29		0.02		−0.32		−13.07	0.000		0.57	1.72
	Self-Efficacy		−0.01		0.00		−0.09		−4.04	0.000		0.70	1.42
	Interpersonal		0.10		0.02		0.09		4.41	0.000		0.69	1.43
	Intrapersonal		−0.05		0.01		−0.06		−2.94	0.003		0.77	1.29

**Table 3:** Stepwise multiple linear regression model (*n* = 1777).

**Energy–Joy**	**Model**	***R***	***R*^2^**	**Corrected *R*^2^**		**Change statistics**							**Durbin–Watson**
						**Standard error of estimation**		**Change in *R*^2^**	**Change in *F***		**Sig. of change in *F***		
	1	0.59	0.35	0.35		0.48		0.35	972.00		0.000		1.94
	2	0.60	0.36	0.36		0.47		0.01	38.94		0.000		
	3	0.61	0.38	0.37		0.47		0.01	36.42		0.000		
	4	0.62	0.38	0.38		0.47		0.00	11.47		0.001		
	5	0.62	0.38	0.38		0.47		0.00	3.98		0.046		
	**Model 5**		**Non-standardized coefficients**				**Standardized coefficients**		***t***	**Sig.**		**Collinearity**	
			***B***		**Standard Error**		**Beta**					***Tol.***	**VIF**
	(Constant)		0.29		0.09				2.97	0.003			
	Mood		0.44		0.02		0.44		18.31	0.000		0.57	1.72
	Stress management		0.13		0.02		0.12		6.33	0.000		0.86	1.16
	Self-efficacy		0.01		0.00		0.11		4.95	0.000		0.70	1.42
	Intrapersonal		0.05		0.01		0.06		2.84	0.004		0.77	1.29
	Interpersonal		0.05		0.02		0.04		1.99	0.046		0.69	1.43
**Fear–Anxiety**	**Model**	***R***	***R*^2^**	**Corrected *R*^2^**		**Change statistics**							**Durbin–Watson**
						**Standard error of estimation**		**Change in *R*^2^**	**Change in *F***		**Sig. of change in *F***		
	1	0.43	0.19	0.19		0.63		0.19	418.20		0.000		1.97
	2	0.50	0.25	0.25		0.60		0.06	148.95		0.000		
	3	0.51	0.26	0.26		0.60		0.00	17.74		0.000		
	4	0.51	0.26	0.26		0.60		0.00	19.89		0.000		
	5	0.52	0.27	0.27		0.60		0.00	6.35		0.012		
	**Model 5**		**Non-standardized coefficients**				**Standardized coefficients**		***t***	**Sig.**		**Collinearity**	
			***B***		**Standard Error**		**Beta**					**Tol.**	**VIF**
	(Constant)		4.39		0.12				34.64	0.000			
	Mood		−0.35		0.03		−0.30		−11.55	0.000		0.57	1.72
	Stress management		−0.33		0.02		−0.27		−12.43	0.000		0.86	1.16
	Self-efficacy		−0.01		0.00		−0.12		−5.05	0.000		0.70	1.42
	Interpersonal		0.16		0.03		0.12		4.92	0.000		0.69	1.43
	Intrapersonal		−0.05		0.02		−0.05		−2.52	0.012		0.77	1.29
**Self-Realization–Satisfaction**	**Model**	***R***	***R*^2^**	**Corrected *R*^2^**		**Change statistics**							**Durbin–Watson**
						**Standard error of estimation**		**Change in *R*^2^**	**Change in *F***		**Sig. of change in *F***		
	1	0.53	0.28	0.28		0.47		0.28	719.94		0.000		1.91
	2	0.58	0.33	0.33		0.46		0.05	133.08		0.000		
	3	0.59	0.35	0.35		0.45		0.01	47.09		0.000		
	**Model 3**		**Non-standardized coefficients**				**Standardized coefficients**		***t***	**Sig.**		**Collinearity**	
			***B***		**Standard Error**		**Beta**					**Tol.**	**VIF**
	(Constant)		4.22		0.09				46.70	0.000			
	Mood		−0.35		0.02		−0.37		−16.26	0.000		0.68	1.47
	Stress management		−0.23		0.02		−0.23		−11.52	0.000		0.86	1.15
	Self-efficacy		−0.01		0.00		−0.15		−6.86	0.000		0.75	1.32
